# Efficient simultaneous production of extracellular polyol esters of fatty acids and intracellular lipids from inulin by a deep-sea yeast *Rhodotorula paludigena* P4R5

**DOI:** 10.1186/s12934-019-1200-3

**Published:** 2019-09-03

**Authors:** Mengqi Wang, Weian Mao, Xiaoxiang Wang, Fengyi Li, Jiming Wang, Zhe Chi, Zhenming Chi, Guanglei Liu

**Affiliations:** 1 College of Marine Life Science, Ocean University of China, Yushan Road, No. 5, Qingdao, 266003 Shandong China; 2 Laboratory for Marine Biology and Biotechnology, Pilot National Laboratory for Marine Science and Technology, No.1 Wenhai Road, Qingdao, 266237 China; 3 Key Laboratory of Biobased Materials, Qingdao Institute of Bioenergy and Bioprocess Technology, Chinese Academy of Sciences, Qingdao, 26601 China

**Keywords:** *Rhodotorula paludigena*, Biosurfactant, Microbial lipid, Biodiesel, Inulin

## Abstract

**Background:**

Polyol esters of fatty acids (PEFA) are a kind of promising biosurfactants and mainly secreted by *Rhodotorula* strains. In addition, some strains of *Rhodotorula* are reliable producers of microbial lipid. Therefore, it is feasible to establish a one step fermentation process for efficient simultaneous production of PEFA and microbial lipids by a suitable *Rhodotorula* strain.

**Results:**

A newly isolated deep-sea yeast, *Rhodotorula paludigena* P4R5, was shown to simultaneously produce high level of intracellular lipid and extracellular PEFA. Under the optimized conditions, it could yield 48.5 g/L of PEFA and 16.9 g/L of intracellular lipid within 156 h from inulin during 10-L batch fermentation. The PEFA consisting of a mixture of mannitol esters of 3-hydroxy C_14_, C_16_ and C_18_ fatty acids with variable acetylation showed outstanding surface activity and emulsifying activity, while the fatty acids of the intracellular lipid were mainly C_16_ and C_18_ and could be high-quality feedstock for biodiesel production.

**Conclusion:**

The deep-sea yeast strain *R. paludigena* P4R5 was an excellent candidate for efficient simultaneous of biosurfactants and biodiesel from inulin. Our results also suggested that the establishment of fermentation systems with multiple metabolites production was an effective approach to improve the profitability.

## Background

Marine yeasts live in harsh environments, and this provides the potential for several unique desirable properties to be used in various industries, including higher osmosis tolerance, higher special chemical productivity and production of industrial enzymes [1]. Therefore, marine yeasts have great potential application in food, pharmaceutical, cosmetic and chemical industries as well as marine culture and environmental protection. The predominant yeasts derived in marine environments are red yeasts, which are primarily members of the genera *Rhodotorula*, *Rhodosporidium* and *Sporobolomyces* [2]. Among these, yeast species of the anamorphic genus *Rhodotorula* have been recognized as a reliable producer of carotenoids, enzymes, microbial oils and biosurfactants [3–5]. Thus, the strains of *Rhodotorula*, especially from marine environments, are of great biotechnological potential and deserve further study and exploration.

Biosurfactants are a structurally diverse group of amphipathic substances molecules synthesized by bacteria, yeasts or fungi [6]. In comparison to chemical surfactants, biosurfactants possess lower toxicity, higher biodegradability and environmental compatibility, and can be produced from regenerated biomass resources and industrial by-products [7]. Therefore, in addition to classical cleaning applications, they have promising applications in environmental protection, crude-oil recovery, food-processing, biofilm prevention and disruption, and in various fields of biomedicine [8–10]. Glycolipids are a group of the most common biosurfactants and mainly consist of sophorolipids, mannosylerythritol lipid, cellobiose lipids, liamocins and polyol esters of fatty acids [4]. Among these, polyol esters of fatty acids (PEFA) are composed of an acetylated (*R*)-3-hydroxy fatty acid esterified through the carboxyl end to a 5 or 6 carbon polyol, typically d-mannitol or d-arabitol, with varying degrees of acetylation [11]. Intriguingly, the PEFA secreting strains are mostly belong to the genus *Rhodotorula* [12]. Specifically, *Rhodotorula babjevae* [13], *Rhodotorula taiwanensis* [14], *Rhodotorula* aff. *paludigena* [15] have been reported to be potential PEFA producers for commercial application. In addition to PEFA, yeast species of the genus *Rhodotorula* have prodigious potential and extensive foreground in intracellular lipids production, known as single cell oils, which serve as precursors for biofuels, oleochemicals and food products. For instance, an oleaginous yeast strain *Rhodotorula toruloides* Y4 could produce high cellular lipid content of over 70% and high cell density of 100 g/L [16].

So far, high production costs are still a main barrier to the commercialization of microbial oils and glycolipids [7]. To overcome this constraint, the establishment of fermentation systems with multiple metabolites production is a promising solution, due to the efficient utilization of fermentation equipment and feedstock [17]. Moreover, both the biosynthesis of glycolipids and intracellular lipids involve fatty acid synthesis pathway, which is enhanced under the conditions of carbon excess and nitrogen limiting (high C:N ratio) [3, 15]. In our previous study, *Aureobasidium melanogenum* 9-1 could simultaneously produce 27.4 ± 0.3 g/L liamocins and 22.6 ± 0.8% (w/w) intracellular lipids within 168 h [18]. Many efforts have concentrated on using industrial waste and raw biomass materials as low-cost carbon sources, such as molasses, whey, industrial fats, glycerol, cassava and lignocellulose [19]. In addition, inulin, a linear polysaccharide (β-2,1-linked d-fructose residues terminated by a glucose residue) presented as a storage carbohydrate in plants such as chicory, Jerusalem artichoke and dahlia, has also received attention as a renewable non-food biomass resource for the production of ethanol, pullulan, single cell oil, citric acid and other chemicals [20–22]. However, the utilization efficiency of inulin depends on the native or heterogenous inulinase activity of yeast strains.

In the present study, a deep-sea yeast strain *Rhodotorula paludigena* P4R5 was isolated and identified. This strain possessed high-level inulinase activity, and could simultaneously produce high quantities of extracellular PEFA and intracellular lipids from inulin. The produced PEFA and intracellular lipids were analyzed and characterized, indicating the potential of the strain P4R5 for the industrial production of biosurfactant and biodiesel.

## Results and discussion

### Isolation and identification of high biosurfactant and lipid producing yeasts from deep sea sediments

**Fig. 1:**
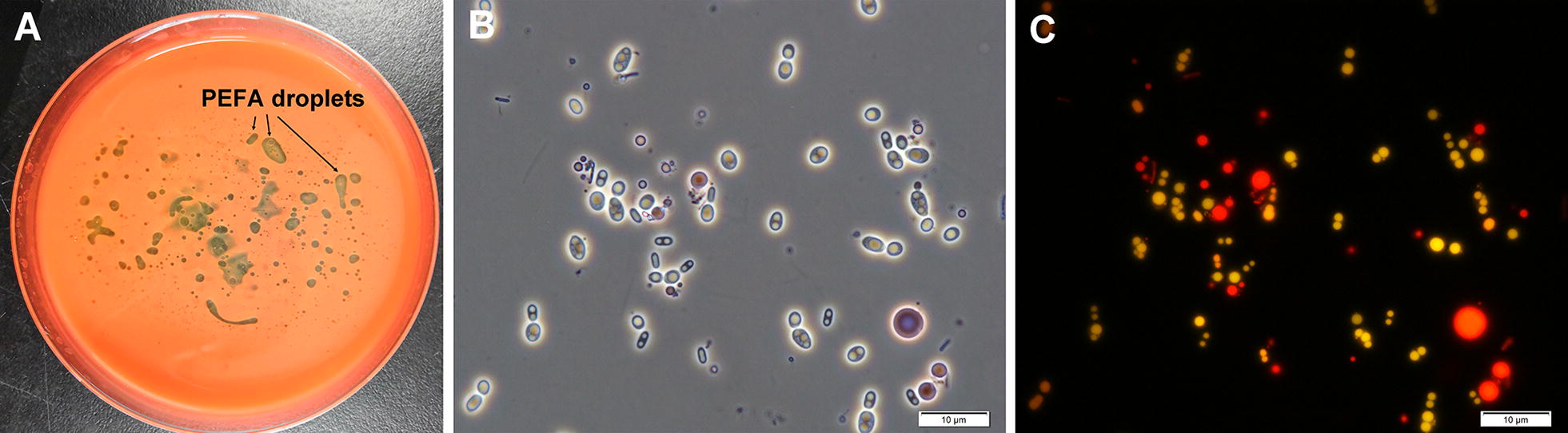
The yellowish and hydrophobic PEFA droplets secreted by the strain P4R5 in the culture (**A**). Intracellular lipid particles (yellow ones) and extracellular liamocin particles (red ones) produced by the strain P4R5 was observed under a phase-contrast microscope (**B**) and a fluorescence microscope (**C**). The strain R4P5 was aerobically grown in the PEFA screening medium at 28 °C for 144 h, and the harvested culture broth was stained with Nile Red

**Fig. 2:**
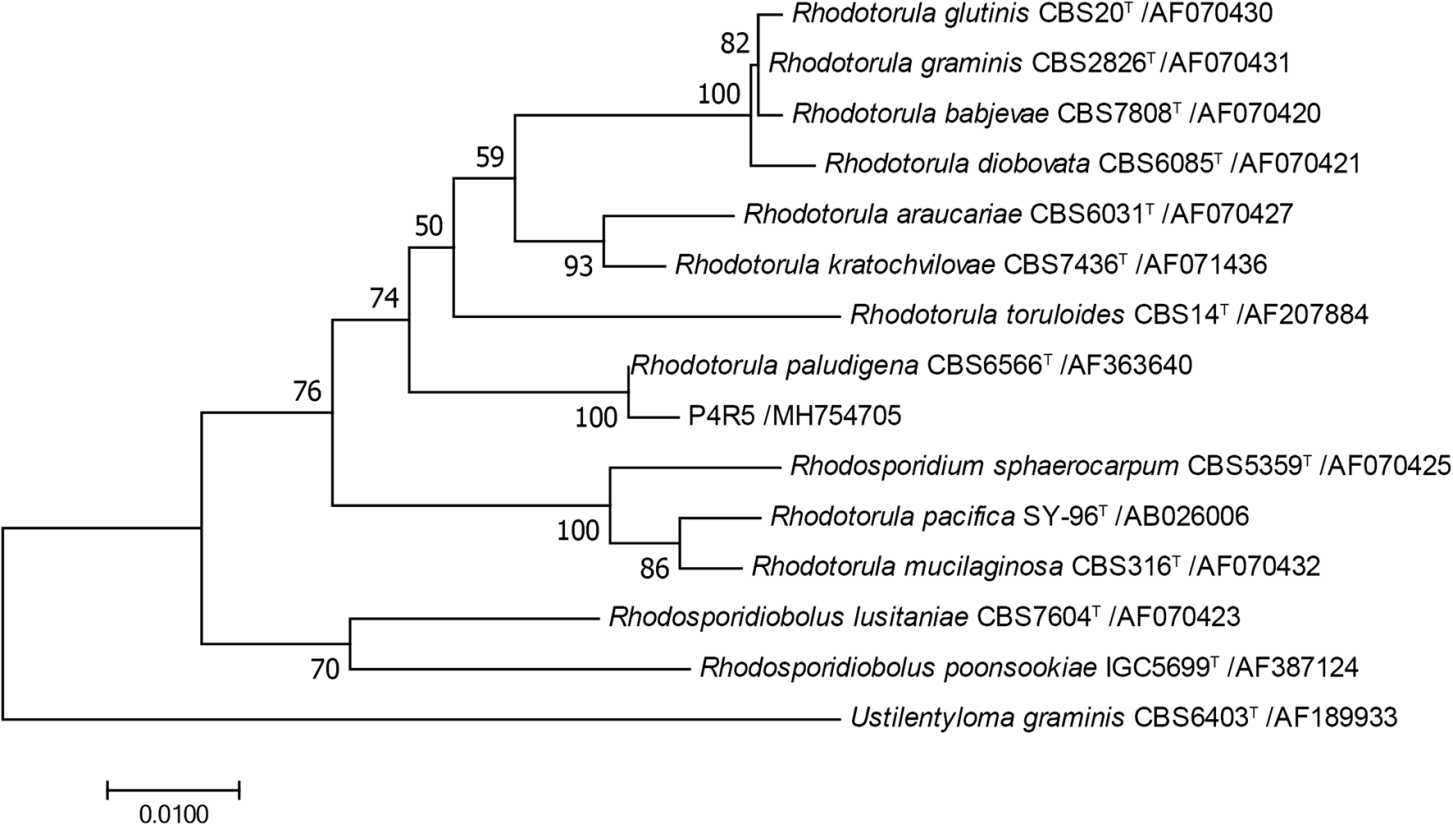
The phylogenetic trees of the yeast strain P4R5 and other yeast relatives based on a neighbor-joining analysis of D1/D2 26S rDNA sequences. Bootstrap values at the notes are from 1000 replicates. ^T^ = type strain

Forty-two strains of yeasts were isolated from sediment samples collected at a depth of 4067 m in the South Pacific (18°29′S, 129°31′W). On the basis of morphological and physiological characteristics [23] and molecular identification, nineteen strains obtained were found to belong to the genus of *Rhodotorula* (Additional file 1: Table S1). Similarly, in a previous study, the proportions of red yeasts among the total yeasts isolated from deep-sea sediments (≥ 2000 m) in the northwest Pacific Ocean was about 50%, suggesting that red yeasts were the predominant yeasts detected in deep-sea environments [2]. Subsequently, to investigate the abilities of intracellular lipids and PEFA production, these nineteen strains were cultivated on the PEFA screening medium, and the yields of biomass, intracellular lipids and PEFA were analyzed within 144 h as described in “Materials and methods” and summarized in Additional file 1: Table S1. Specifically, the strain P4R5 could produce distinguishingly numerous hydrophobic droplets, displaying a yellowish and viscous appearance (Fig. 1A), and the morphologic investigation of the P4R5 culture under fluorescence microscopy via Nile red staining also revealed the presence of not only yellow fluorescent intracellular lipids but also red fluorescent lipid molecules in extracellular compartment, indicating the ability of simultaneous production of extracellular PEFA and intracellular lipids (Fig. 1B, C). Furthermore, intracellular lipid content and extracellular PEFA titer produced by strain P4R5 within 144 h were 48.9% (w/w) and 12.7 g/L, respectively. Based on these particularity, the strain P4R5 was selected for further study. The sequence of the D1/D2 region of the 26S rRNA gene of the strain P4R5 (Accession number: MH754705) showed 99% with 99  % that of *Rhodotorula paludigena* CBS6566^T^ (type strain). Furthermore, the topology of the phylogenetic tree in Fig. 2 confirmed that the yeast strain P4R5 belonged to the species *R. paludigena*. So far, the vast majority of the PEFA secreting yeast strains, mostly belonging to genus *Rhodotorula*, were isolated from plant surfaces [12]. Therefore, *R. paludigena* P4R5 isolated from deep sea was a new producer of extracellular PEFA and intracellular lipids.

### Analysis and characterization of PEFA and intracellular lipids produced by *R. paludigena* P4R5

Each PEFA-secreting yeast produced a mixture of structurally similar PEFA, differing in the type of polyols attached, the degree of acetylation in the sugar alcohol, and the chain length of the 3-hydroxy fatty acid [4]. In order to characterize the fatty acid composition of PEFA and intracellular lipids in this study, the intracellular lipids and the extracellular PEFA produced from the strain P4R5 on the PEFA screening medium within 144 h were extracted and subjected to transmethylation for GC/MS analysis. As shown in Table 1, it was found that over 88% of the intracellular fatty acids from the strain P4R5 were C_16:0_, C_18:0_, C_18:1_ and C_18:2_, especially C_18:1_ (35.93%). This composition was similar to those of the other oleaginous yeast lipids reported [22, 24, 25], and such yeast oils could be used as high-quality feedstock in biodiesel production that requires C_16_–C_18_ fatty acids [26]. For extracellular PEFA, the GC/MS analysis indicated the presence of three major hydroxyl fatty acids of 3-OH-C_14:0_ (4.40%), 3-OH-C_16:0_ (70.51%) and 3-OH-C_18:0_ (15.97%), in addition to a small amount of 2-hexadecenoic acid and 2-octadecenoic acid, which were probably resulted from the dehydration of the corresponding 3-hydroxyl fatty acids during the methyl esterification process (Table 1). Notably, 3-hydroxyhexadecanoic acid (3-OH-C_16:0_) and 3-hydroxyoctadecanoic acid (3-OH-C_18:0_) were also detected in the intracellular components. This suggested that the hydroxylation of fatty acids in the strain P4R5 reacted inside the cell, and this process was probably catalyzed an cytochrome P450 monooxygenase bound to the endoplasmic reticulum membrane according to the reported sophorolipid synthesis pathway and the genetic studies in the *Rhodotorula* strains [12, 27]. Based on the results above, the chain lengths of the 3-hydroxy fatty acids of the PEFA from the strain P4R5 were C14, C16 and C18.

**Table 1:** Fatty acid composition of the intracellular lipids and extracellular PEFA produced by *R. paludigena* P4R5 cultivated on PEFA screening medium for 144 h

Composition	Percentage (%)	
	Intracellular fatty acid	Extracellular fatty acid
Tetradecanoic acid (C_14:0_)	1.75	–
3-Hydroxytetradecanoic acid (3-OH-C_14:0_)	–	4.40
Hexadecanoic acid (C_16:0_)	31.92	–
2-Hexadecenoic acid (C_16:1_)	0.55	7.50
3-Hydroxyhexadecanoic acid (3-OH-C_16:0_)	6.51	70.51
Octadecanoic acid (C_18:0_)	6.13	–
9-Octadecenoic acid (C_18:1_)	35.93	–
9,12-Octadecadienoic acid (C_18:2_)	12.26	–
9,12,15-Octadecatrienoic acid (C_18:3_)	2.57	–
2-Octadecenoic acid (C_18:1_)	–	1.62
3-Hydroxyoctadecanoic acid (3-OH-C_18:0_)	1.29	15.97
Eicosanoic acid (C_20:0_)	0.20	
Docosanoic acid (C_22:0_)	0.43	
Tetracosanoic acid (C_24:0_)	0.45	

**Fig. 3:**
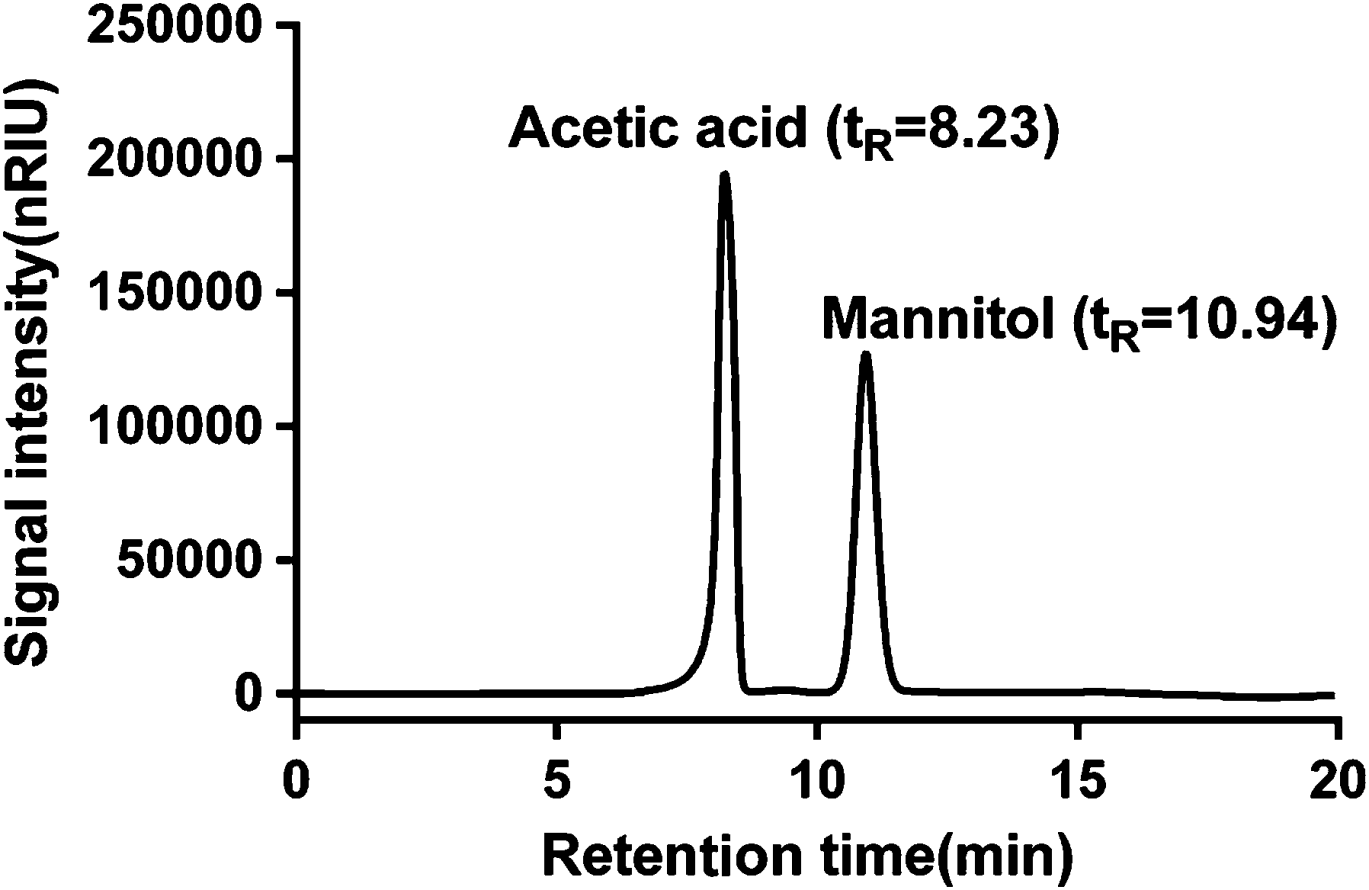
HPLC analysis of polyols and acetic acid. According to the retention time of the standard substances, acetic acid and mannitol were detected

To analyze the type of polyols and degree of acetylation, the polyols and acetic acid from the alkaline hydrolysate of the extracellular PEFA were determined by HPLC. According to the retention time of the standard substances, acetic acid and mannitol were detected with a molar ratio of 4.2 (Fig. 3). This result indicated that the polyol group of the PEFA from strain P4R5 was only mannitol, and the average degree of acetylation in the mannitol was 3.2, considering the acetylation in the 3-hydroxy of fatty acid in all yeast-secreting PEFA congeners reported [4, 15, 17]. It has demonstrated that the acetylation of polyol groups on impacted the hydrophilic-lipophilic balance of PEFA, and hyper-acetylated PEFA having a low surfactant activity [14]. Therefore, the PEFA produced by *R. paludigena* P4R5 with the moderate acetylation could be effective and worthy of exploitation as a new biosurfactant.

The PEFA produced by *R. paludigena* P4R5 were further analyzed by liquid chromatography-electrospray ionization-mass spectrometry (LC–ESI–MS). The mass spectral ions were identified by calculation of elemental composition and comparison with available literature [4, 15, 17]. The result in Table 2 revealed the presence of at least 13 major fractions with molecular mass range of *m/z* 501.26–711.30, including acetylated 3-hydroxy hydroxytetradecanoic acid esterified to d-mannitol with 1–4 acetylation, acetylated 3-hydroxy hydroxyhexadecanoic acid esterified to mannitol with 0–5 acetylation, and acetylated 3-hydroxy hydroxyoctadecanoic acid esterified to mannitol with 0 and 2–4 acetylation. These results corroborated the findings of the fatty acid composition (Table 1) and the polyols type analysis (Fig. 3).

**Table 2:** Identification of PEFA produced by *R. paludigena* P4R5 cultivated on PEFA screening medium for 144 h

PEFA no.	Chemical formula	m/z [M + Na]^+^	Description
1	C_24_H_44_O_10_	515.28	Acetylated C14:0 3-hydroxy fatty acid esterified to mannitol with 1 acetylation
2	C_26_H_46_O_11_	557.28	Acetylated C14:0 3-hydroxy fatty acid esterified to mannitol with 2 acetylations
3	C_28_H_48_O_12_	599.31	Acetylated C14:0 3-hydroxy fatty acid esterified to mannitol with 3 acetylations
4	C_30_H_50_O_13_	641.34	Acetylated C14:0 3-hydroxy fatty acid esterified to mannitol with 4 acetylations
5	C_24_H_46_O_9_	501.26	Acetylated C16:0 3-hydroxy fatty acid esterified to mannitol with 0 acetylation
6	C_26_H_48_O_10_	543.33	Acetylated C16:0 3-hydroxy fatty acid esterified to mannitol with 1 acetylation
7	C_28_H_50_O_11_	585.32	Acetylated C16:0 3-hydroxy fatty acid esterified to mannitol with 2 acetylations
8	C_32_H_54_O_13_	669.38	Acetylated C16:0 3-hydroxy fatty acid esterified to mannitol with 4 acetylations
9	C_34_H_56_O_14_	711.30	Acetylated C16:0 3-hydroxy fatty acid esterified to mannitol with 5 acetylations
10	C_26_H_50_O_9_	529.34	Acetylated C18:0 3-hydroxy fatty acid esterified to mannitol with 0 acetylation
11	C_30_H_54_O_11_	613.40	Acetylated C18:0 3-hydroxy fatty acid esterified to mannitol with 2 acetylations
12	C_32_H_56_O_12_	655.32	Acetylated C18:0 3-hydroxy fatty acid esterified to mannitol with 3 acetylations
13	C_34_H_58_O_13_	697.39	Acetylated C18:0 3-hydroxy fatty acid esterified to mannitol with 4 acetylations

In conclusion, the PEFA produced by *R. paludigena* P4R5 were found to be a mixture of mannitol esters of 3-hydroxy fatty acids with variable acetylation. This result was basically in correlation with the composition identified in other strains of *Rhodotorula* yeast such as *R. babjevae* Y-SL7, *R.* aff. *paludigena* UCDFST, *R. babjevae* UCDFST 04-877 and *R. taiwanensis* MD1149 [14, 15, 17]. However, in addition to different degrees of acetylation, the major difference was the absence the arabitol esters of 3-hydroxy fatty acids in the PEFA of *R. paludigena* P4R5. This could be attributable to the genetic diversification of different strains.

### Effect of different concentrations of glucose on PEFA and intracellular lipids production

**Fig. 4:**
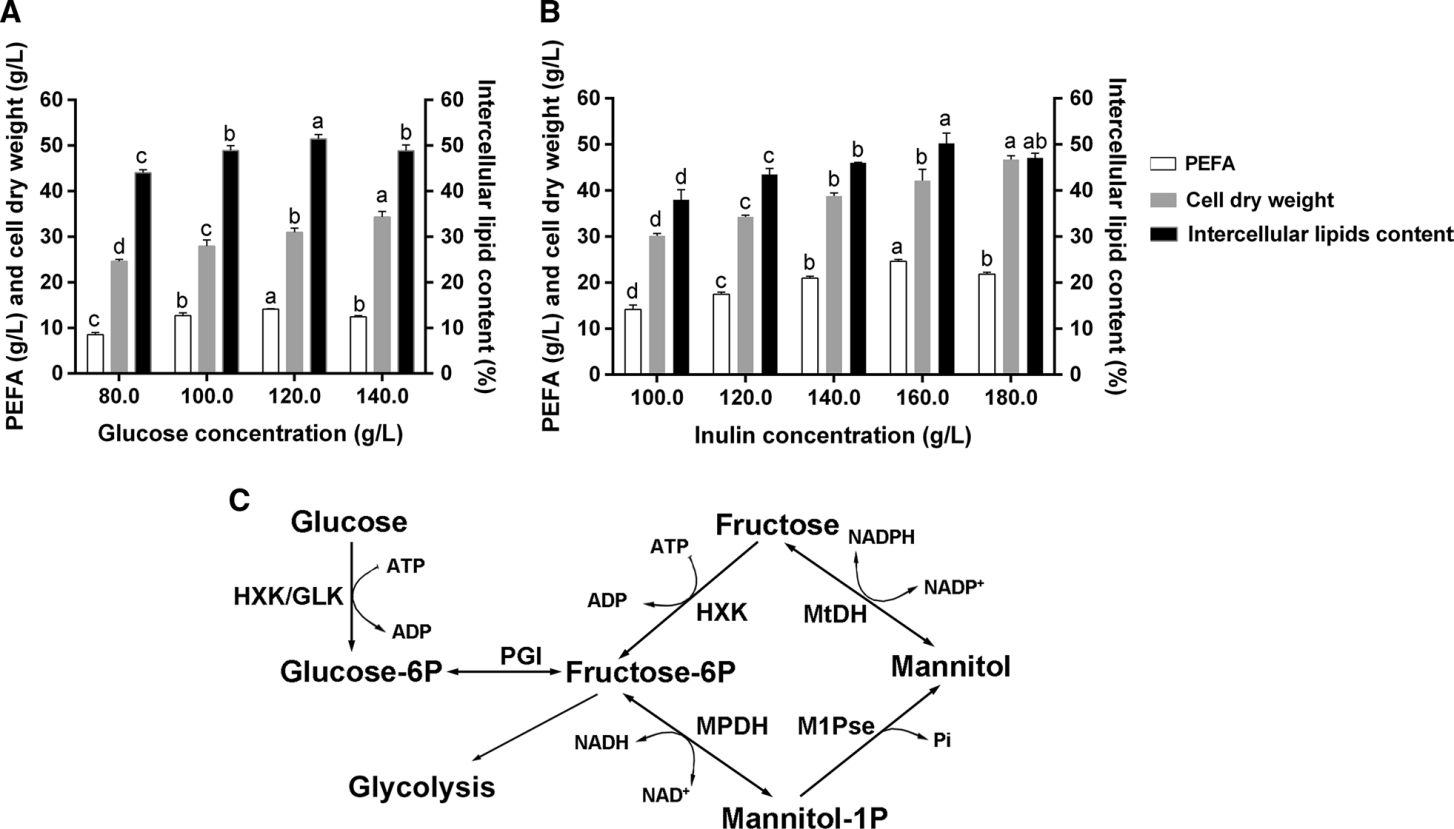
Effects of different concentrations of different concentrations of glucose (**A**) and different concentrations of inulin (**B**) on PEFA production, intercellular lipid content and cell growth. The values were means of three independent determinations. ^a,b,c,d^Mean values with different superscript letters differ significantly and were determined statistically as mentioned in “Materials and methods” (one way ANOVA, Tukey, *P* < 0.05). Separate analysis was done for the groups of PEFA, cell dry weight and intercellular lipids content. **C** Mannitol biosynthetic pathway in fungi. HXK, hexokinase; GLK, glucose kinase; MtDH, mannitol dehydrogenase; MPDH, mannitol-1-phosphate dehydrogenase; M1Pse, mannitol-1-phosphate phosphatase

Unlike some glycolipids consisted of short chain fatty acids, such as liamocins from *Aureobasidium melanogenum* (hydroxy decanoic acid) [18], the fatty acid precursors of PEFA in *R. paludigena* P4R5 were mainly C_16_ and C_18_ (Tables 1, 2). This meant that the synthesis of PEFA and intracellular lipids shared the same fatty acid synthesis pathway, followed by linking with a glycerol or glycosidic molecule to generate triacylgycerols (TAG) or glycolipids, respectively [27]. Therefore, to simultaneously improve the production of PEFA and intracellular lipids, prior efforts should focused on enhancing fatty acid synthesis pathway. For oleaginous yeasts, it has been well documented that a high initial C/N ratio (nitrogen starvation) in the medium is required to boost fatty acid synthesis [3]. Therefore, to improve the PEFA and lipid production by the strain P4R5, it was very important to optimize the glucose concentration in the PEFA screening medium. As shown in Fig. 4A, when the medium contained 120.0 g/L glucose, both the intercellular lipid content and the extracellular PEFA titer reached the highest amount (51.4% and 14.1 g/L, respectively) and cell dry weight was 31.0 g/L within 144 h. Notably, this extracellular PEFA titer (14.1 g/L) were higher than those of other reported *Rhodotorula* strains from glucose at flask level [12], such as *R*. aff. *paludigena* UCDFST 81-84 (12.4 g/L), *R. paludigena* UCDFST 81-492 (11.7 g/L) *R. babjevae* UCDFST 04-830 (8.5 g/L). This suggested that the strain P4R5 in this study had an superior capacity of PEFA production. With regard to C/N molar ratio, the initial value was 89.5 in the optimum medium for PEFA production, considering the presence of 1.0 g/L yeast and 0.2 g/L ammonium sulfate and 120.0 g/L glucose. In a previous study, the strain *R. babjevae* Y-SL7 reached the highest production of intra and extracellular lipids (3.5 and 4.4 g/L, respectively) at a C/N molar ratio of 100 [17]. These results verified that the sharp initial C/N ratio enhanced fatty acid synthesis and further pushed the formation of TAG and PEFA. However, as shown in Fig. 4A, when the glucose concentration increased to 140.0 g/L, the production of both the intercellular lipids and the PEFA began to drop. The trend was likely ascribed to the effect of osmotic stress caused by high concentration of glucose.

### The advantages of inulin over glucose for PEFA production

**Fig. 5:**
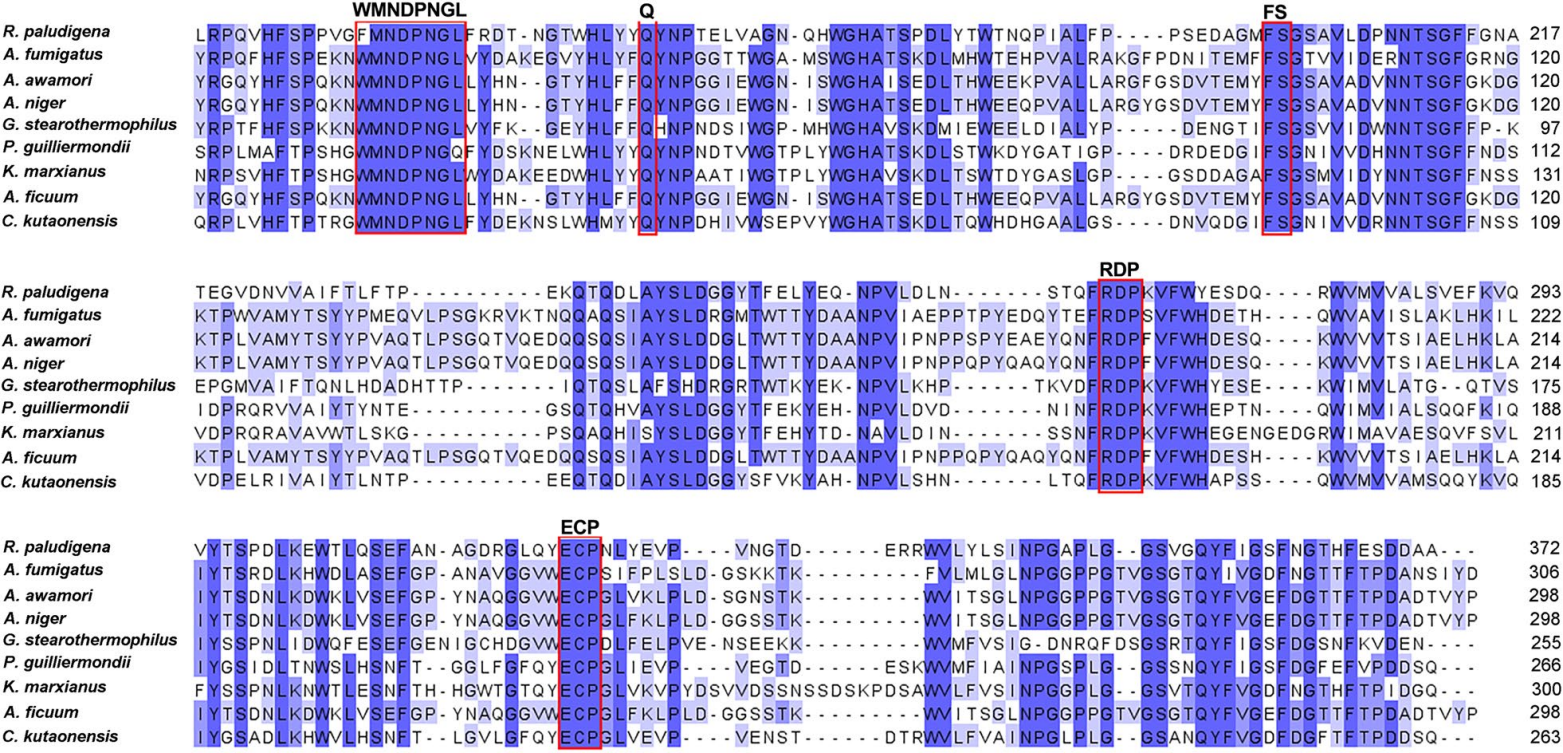
Multiple alignment of the deduced amino acid sequence of the cloned *RpINU* gene with the published amino acid sequences of the exo-inulinases from *Aspergillus fumigatus* (EAL86253), *Aspergillus awamori* (CAC44220), *Aspergillus niger* (AF007559), *Geobacillus stearothermophilus* (BAC45010), *Meyerozyma guilliermondii* (ABW70125), *Kluyveromyces marxianus* (AAN32611), *Aspergillus ficuum* (ADM21204), *Candida kutaonensis* (CCF77887). The alignment was performed using Clustal X 2.0

In addition to glucose, inulin also has received considerable attention as a renewable and competitive non-food biomass resource for microbial lipids production by oleaginous yeasts [20, 22]. For examples, the yeast strains *R. toruloides* 2F5 and *Pichia guilliermondii* Pcla22 could produce 70.36% and 60.6% (w/w) of lipid based on cell dry weight from inulin due to their native exo-inulinase genes, respectively [24, 25]. However, the data available in literature scarcely involved the PEFA production from inulin. In this study, the strain P4R5 could produce inulinase activity of 34.6 U/mL within 72 h in the inulinase production medium, suggesting its potential of direct conversion of inulin into PEFA and cell lipid. To provide further evidence, the inulinase gene of the strain *R. paludigena* P4R5 was cloned and sequenced. The obtained inulinase gene (*RpINU*) had 2068 bp and four exons encoding a protein with 618 amino acids (Accession number: MH924353). Sequence alignment in Fig. 5 revealed that the amino acids deduced from the *RpINU* gene contained the conserved motifs of MNDPNGL, Q, FS, RDP and ECP, which were presented as characterized motifs in exo-inulinases [20]. According to the catalytic mechanism reported previously, the residues Asp^141^ and Glu^320^ of the deduced amino acids acted as a nucleophile and an acid/base catalyst, respectively. These results confirmed that the yeast strain P4R5 indeed had the *RpINU* gene encoding an exo-inulinase.

Subsequently, effect of different concentrations of inulin on PEFA and intracellular lipids production was investigated. As shown in Fig. 4B, when the medium contained 120.0 g/L inulin, the strain P4R5 could produce the PEFA of 17.4 g/L, which was significantly higher than the analogue (14.1 g/L) when the medium contained the optimum glucose concentration of 120.0 g/L (t-test, *P *< 0.01) (Fig. 4A). Furthermore, Fig. 4B indicated that the amounts of PEFA and intracellular lipids were raised progressively with increasing of inulin supply, and reached their maximum of 24.6 g/L and 21.1 g/L (lipids content of 50.2% (w/w)) within 144 h, respectively, at a inulin concentration of 160.0 g/L. These results indicated that inulin was a kind of more superior carbon resource than glucose for the PEFA yield in the strain P4R5. According to the previous literatures and the structure analyzed above (Table 2 and Fig. 3) [12, 27], the PEFA biosynthetic pathway in *R. paludigena* P4R5 is probable that the formation of the fatty acid portion of PEFA undergoes a similar biosynthetic pathway to that of triacylglycerol, followed by hydroxylation and esterification with a polyol group of mannitol. For fatty acid synthesis, inulin and glucose share the same metabolic pathways, because the inulin hydrolysis products are mainly fructose and a small amount glucose [20]. However, the mannitol synthesis initiated by fructose is different from that initiated by glucose, on the basis of the reported biosynthetic pathway in fungi [28]. Specifically, as shown in Fig. 4C, fructose can be directly converted into mannitol by mannitol dehydrogenase (MtDH) at the cost of a molecule of NADPH, while glucose is converted into mannitol in a four-step process that includes hexokinase (HXK), phosphoglucose isomerase (PGI), mannitol-1-phosphate dehydrogenase (MPDH) and mannitol-1-phosphate phosphatase (M1Pse). This meant that the conversion of glucose into mannitol consumes an extra molecule of ATP compared with that of fructose (Fig. 4C). Thus, it could be concluded that inulin was more efficient than glucose for mannitol synthesis in yeast cells. Therefore, utilizing inulin as carbon resource for PEFA production, the improvement of the PEFA yield was probably ascribed to the enhanced efficiency of mannitol synthesis. This conclusion showed good agreements with a previous study that the PEFA yield of the strain *R*. aff. *paludigena* UCDFST 81-84 using sucrose was higher than that obtained with glucose [12]. Because similar to inulin, the hydrolysis products of sucrose are also fructose and glucose. Besides, another probable reason was that inulin caused an lower osmotic stress on yeast cells than glucose under the same concentration. In conclusion, inulin, a promising alternative to glucose, exhibited significant advantages as a carbon source in PEFA production.

### PEFA and intracellular lipids production from inulin in a 10-L batch bioreactor

**Fig. 6:**
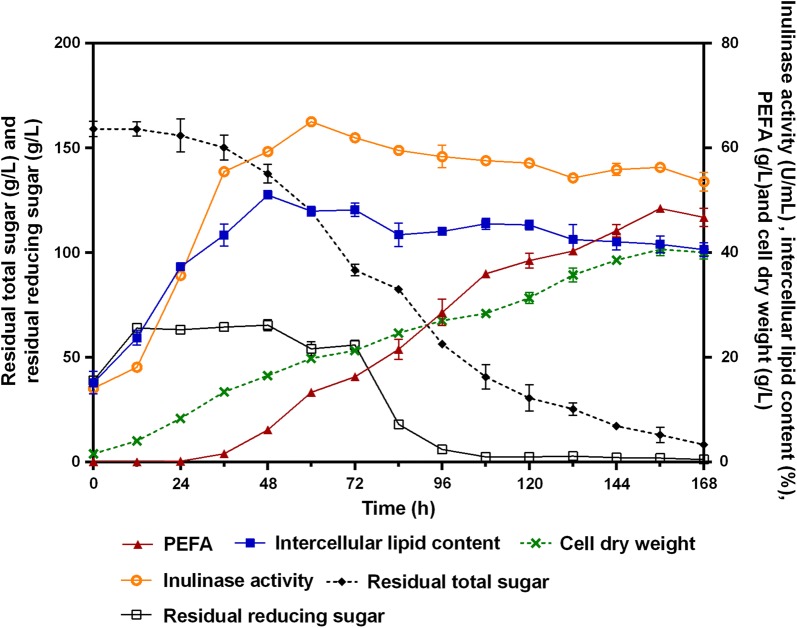
The time course of the extracellular PEFA production, cell growth, intracellular lipid production, inulinase activity, residual reducing sugar and residual total sugar during 10-L fermentation of the strain R4P5. The values were means of three independent determinations

Taking into account high levels production of intracellular lipids and extracellular PEFA, the strain *R. paludigena* P4R5 was selected for larger-scale work using the optimized PEFA production medium containing inulin. As shown in Fig. 6, the inulinase activity reached 65.0 ± 0.7 U/mL within 60 h, which was much more than any other oleaginous yeast strains reported so far, such as *R. toruloides* 2F5 (7.5 U/mL) [25] and *P. guilliermondii* Pcla22 (11.5 U/mL) [24]. This allowed the strain P4R5 to efficiently produce PEFA and intracellular lipids from inulin. For intracellular lipids, during the first 48 h, its amount increased exponentially to reach 51.1% (w/w). Thereafter, its accumulation fell gradually to about 40% (w/w) after 168 h of culture. In contrast, the production of PEFA began at 36 h, and its titer was raised dramatically to achieve a maximum of 48.5 ± 0.2 g/L within 156 h, with cell dry weight and oil content of 40.6 ± 1.2 g/L and 41.6 ± 1.6%, respectively (Fig. 6). These results suggested that, in addition to the newly synthesized fatty acids, the storage lipid also could be mobilized for the PEFA synthesis. In the meantime, the lipid production was accompanied by a massive consumption of inulin, and 5.1% residual total sugar remained at the end of the fermentation period (Fig. 6). Finally, 0.32 g/g of extracellular PEFA and 0.11 g/g of the intracellular lipids were generated at 0.31 and 0.11 g/h/L productivity levels within 156 h, respectively. Previously, several successful attempts made to simultaneously produce extracellular PEFA and intracellular lipids using *Rhodotorula* strains in the bioreactor level, indicating the PEFA yields ranging from 12.3 to 31.2 g/L [12]. For example, in a 7-L bioreactor the yeast strain *R.* aff. *paludigena* UCDFST 81-84 could secret 20.9 g/L PEFA and produced 8.8 g/L TG, while the yeast strain *R. babjevae* UCDFST 04-877 could produce 11.2 g/L PEFA and 18.5 g/L TG, with overall glucose conversion rates of 0.24 and 0.22 g_(total lipid)_/g_(glucose)_, respectively [15]. This demonstrated that the deep-sea strain *R. paludigena* P4R5 used in this study was a prominent candidate for the production of PEFA and intracellular lipids on a large scale in industry because of its high PEFA yield, conversion rate and productivity.

### Physicochemical properties of the PEFA produced by *R. paludigena* P4R5

**Fig. 7:**
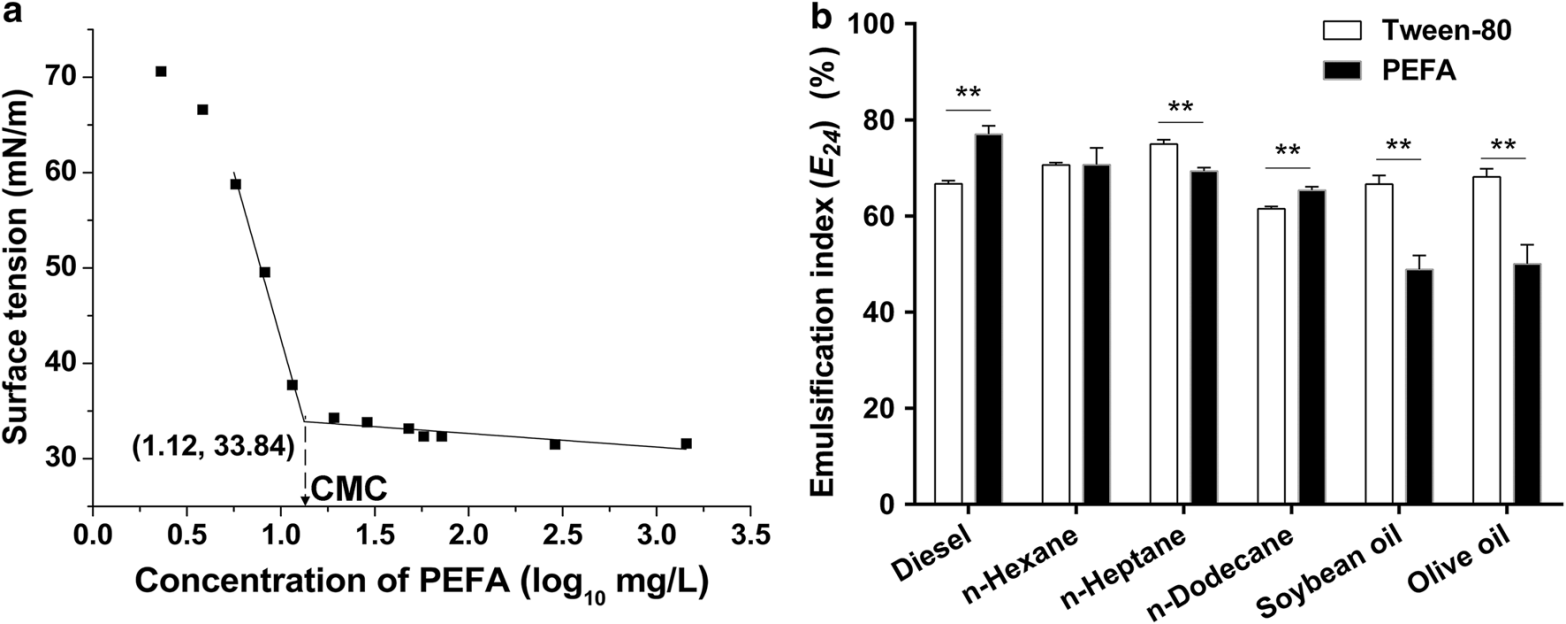
**a** Surface tension as a function of biosurfactant concentration. CMC represents critical micelle concentration. **b** The emulsification activity of the PEFA produced by strain R4P5 on different hydrophobic substrates and comparing with Tween-80. The values were means of three independent determinations. Means between the groups of Tween-80 and PEFA were compared and analyzed using t-test (*P < 0.05, **P < 0.01)

Biosurfactants reduce the surface (liquid–air) and interfacial (liquid–liquid) tension between two dissimilar phases and allow them to mix and interact more easily. The surface tension decreases gradually with increasing the concentration of biosurfactants until the critical micelle concentration (CMC) is obtained [7]. At concentrations above the CMC, biosurfactant molecules associate to form micelles, bilayers and vesicles, and the surface tension remains almost constant [7]. Figure 7a showed the plotting of surface tension versus logarithmic concentration (mg/L) of PEFA produced by the strain P4R5. The surface tension of pure water decreased gradually with increasing PEFA concentration to 33.84 mN/m, with a CMC value of 13.18 mg/L. So far, CMC values at a wide range from 5 to 386 mg/L have been reported for biosurfactants [29]. This suggested that the PEFA produced by the strain P4R5 was efficient, considering that surfactant of low concentration was enough to decrease the surface tension. Generally, the most active biosurfactants can lower the surface tension of water from 72 to approximately 30 mN/m [7]. With regard to the PEFA produced by *Rhodotorula* spp., the reported minimum surface tensions ranged from 30.4 to 35 mN/m [12–14]. For example, PEFA mixtures produced by *R*. aff. *paludigena* UCDFST 81-84 with a minimum surface tension of 33.3 mN/m showed antifoam activity comparable to commercial antifoaming agents used in the brewing industry. In another study, the biosurfactant produced by *R. babjevae* YS3 with a minimum surface tension of 32.6 mN/m exhibited antifungal activity against a considerably broad group of pathogenic fungi [13], while the PEFA from *R. babjevae* Y-SL7 showed a cytotoxic effect against different cancer cell lines [17]. Therefore, the PEFA produced by the strain P4R5 with a surface tension of 33.84 mN/m at the CMC value of 13.18 mg/L has promising application prospects.

In addition, the emulsification activity of the PEFA produced by the strain P4R5 was also investigated on different hydrophobic substrates and compared with commercial biosurfactants Tween 80. As shown in Fig. 7b, the PEFA possessed higher emulsification indexs towards diesel (77.0%) and alkanes (*E*_24_ values ranged from 70.7 to 63.4%) than those towards fatty acid esters. Intriguingly, the emulsification ability of the PEFA was significantly better than that of Tween 80 in the presence of diesel, indicating that the PEFA from *R. paludigena* P4R5 had an environmental interest in the biodegradation of polluting fossil fuels. Similarly, a recent publication by Guerfali et al. [17] revealed the PEFA from the strain *R. babjevae* Y-SL7 showed higher emulsification indexs towards diesel and mineral oil than those towards vegetable oils. In another study, a biosurfactant produced by *Halomonas* sp. MB-30 exhibited very high emulsification activity towards crude oil and kerosene, thus it could be used for in situ biosurfactant-mediated enhanced oil recovery process [30].

### Properties of the biodiesel prepared using intracellular lipids produced by *R. paludigena* P4R5

**Fig. 8:**
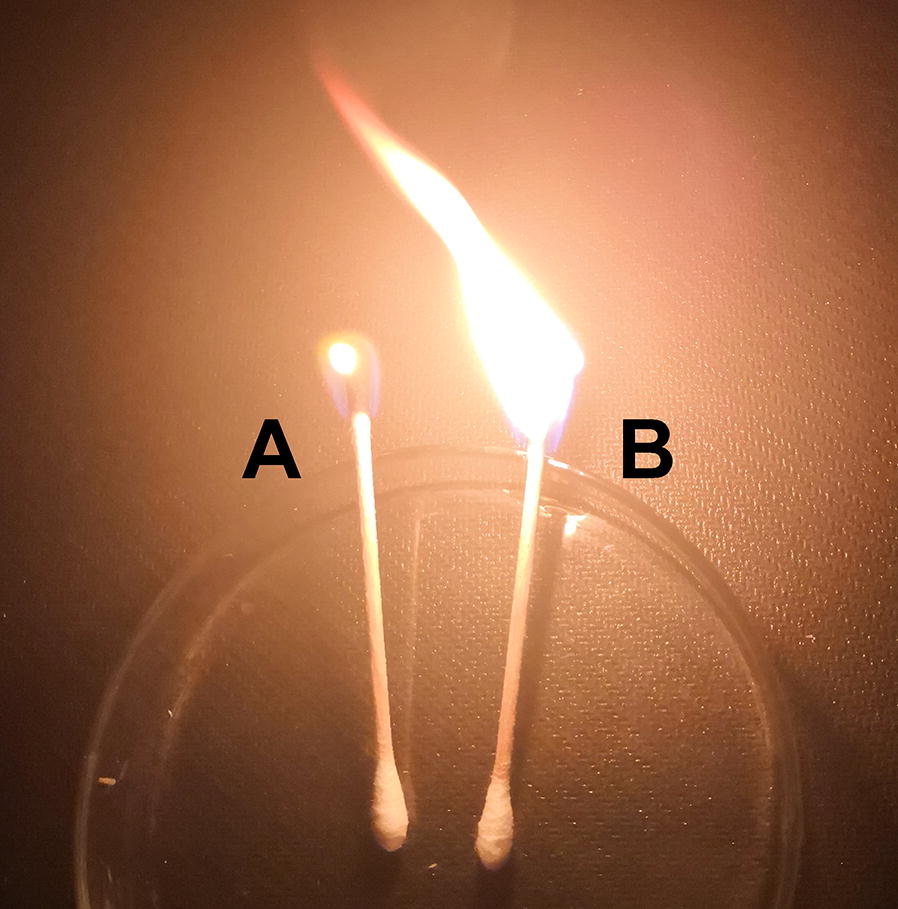
The burning of the prepared biodiesel from the intracellular lipids of the strain P4R5 (B) and the control without biodiesel (A)

Biodiesel have gained much attention because of its non-toxicity, renewability, biodegradability, inherent lubricity, low or no sulfur content, high flash point, and the reduction of most regulated exhaust emissions [31]. In this study, in addition to PEFA, the strain *R. paludigena* P4R5 could simultaneously accumulate large amount of intracellular lipids (Fig. 6), which were mainly C_16_–C_18_ fatty acids (Table 1) and could be used for biodiesel production. Therefore, the intracellular lipids obtained from the cells of *R. paludigena* P4R5 were converted into biodiesel. As shown in Fig. 8, it was found that the prepared biodiesels could be burnt well. Considering that direct measurement of fuel properties of biodiesel is quite complex with high cost, high error in reproducibility and requiring a considerable amount of fuel sample, prediction models and mathematical equations have been developed to predict biodiesel properties from fatty acid methyl ester composition [32, 33]. In the present study, all the properties of the prepared biodiesel listed in Table 3, including viscosity, specific gravity, cloud point, cetane number, iodine number and higher heating value (HHV), were met the US and European specifications for biodiesel. Similar to other biodiesel prepared from microbial lipids, the biodiesel obtained in this study showed lower iodine value and higher cloud point than those of the biodiesel prepared from vegetable oils (soybean and sunflower in Table 3), duo to lower degree of unsaturation. Specifically, the total percentages of C_18:1_, C_18:2_ and C_18:3_ fatty acids in soybean oil and sunflower oil were 84.09% and 90.62 [31], respectively, while the analogue in the intracellular lipids produced by *R. paludigena* P4R5 was 50.76 (Table 1). Although higher cloud point limits low-temperature application of biodiesel, but lower iodine values means less sensitive to oxidation and more stable. In addition, the use of edible vegetable oils is associated with serious environmental problem, such as deforestation, destruction of vital soil resources and consumption of much of the arable land. Therefore, lipids from oleaginous yeasts including *R. paludigena* P4R5 may serve as candidates for biodiesel production.

**Table 3:** Properties of biodiesel from the yeast strain *R. paludigena* P4R5, soy oil, corn oil, US biodiesel, and EU biodiesel standards

	Viscosity (mm^2^/s)	Iodine value	Specific gravity	Cetane no. (min)	HHV (MJ/kg)	Cloud point (°C)	Ref
*R. paludigena* P4R5^a^	4.77	63.81	0.8764	58.29	39.74	10.82	This study
Soybean	4.15	117.7	0.882	44.7	40.25	0.5	[30]
Sunflower	4.26	128.7	0.869	45.7	35.74	0	[30]
US biodiesel ASTM D6751-08	1.9–6.0	–	–	47	–	–	[35]
Europe biodiesel (EN 14214)	3.5–5.0	120 max	0.86–0.9	51	–	–	[35]

^a^The yeast lipids for biodiesel preparation was produced by *R. paludigena* P4R5 from inulin within 156 h during a 10-L batch fermentation

## Conclusions

In this study, a yeast strain *R. paludigena* P4R5 isolated from deep-sea sediment showed promising biotechnological properties, such as outstanding inulinase activity and the ability of simultaneous high-level production of intracellular microbial lipid and extracellular PEFA. During 10-L batch fermentation, the strain P4R5 could produce 48.5 g/L of PEFA and 16.9 g/L of intracellular lipid from inulin with the productivity levels of 0.31 and 0.11 g/h/L, indicating that the fermentation system for multiple metabolites production was efficient. Structure and physicochemical properties analysis revealed that the PEFA produced by the strain P4R5 was composed of a mixture of mannitol esters of 3-hydroxy C_14_, C_16_ and C_18_ fatty acids with variable acetylation, and showed promising application prospects as biosurfactant due to its good surface activity and emulsifying activity. In addition, the intracellular lipids, which were mainly C_16_–C_18_ fatty acids, could be used as high-quality feedstock for biodiesel production. These results suggested that the deep-sea yeast strain *R. paludigena* P4R5 was an excellent candidate for industrial production of biosurfactants and biodiesel.

## Materials and methods

### Yeast strain and media

Yeast strains were isolated from sediment samples from a location of 18°29′S, 129°31′W at a depth of 4067 m in the South Pacific at station BC04 during the 46th Chinese COMRA (China Ocean Mineral Resources Research & Development Association) cruise. YPD medium for yeast growth contained 20.0 g/L glucose, 10.0 g/L yeast extract and 20.0 g/L peptone. PEFA screening medium consisted of 100.0 g/L glucose, 1.0 g/L yeast extract, 0.2 g/L (NH_4_)_2_SO_4_, 7.0 g/L KH_2_PO_4_, 2.5 g/L Na_2_HPO_4_, 1.5 g/L MgSO_4_·7H_2_O, 0.15 g/L CaCl_2_, 0.15 g/L FeCl_3_∙6H_2_O, 0.02 g/L ZnSO4·7H_2_O, and 0.02 g/L MnSO_4_∙H_2_O. Inulinase production medium was composed of 20.0 g/L inulin, 10.0 g/L yeast extract and 20.0 g/L peptone. The optimized medium for PEFA production consisted of 160.0 g/L inulin (Pioneer Biotech Co. Ltd., Xian, China), 1.0 g/L yeast extract, 0.2 g/L (NH_4_)_2_SO_4_, 7.0 g/L KH_2_PO_4_, 2.5 g/L Na_2_HPO_4_, 1.5 g/L MgSO_4_·7H_2_O, 0.15 g/L CaCl_2_, 0.15 g/L FeCl_3_∙6H_2_O, 0.02 g/L ZnSO4·7H_2_O, and 0.02 g/L MnSO_4_∙H_2_O.

### DNA extraction, PCR amplification, DNA sequencing, and phylogenetic analysis

The genomic DNAs from the isolated deep-sea yeasts were extracted using a TIANamp Yeast DNA Kit (TIANGEN, Beijing, China). Amplification and sequencing of D1/D2 26S rDNA sequences from this yeast strain was performed according to the methods described by Wang et al. [25] using the primers NL-1:5′-GCATATCAATAAGCGGAGGAAAAG-3′ and NL-4:5′-GGTCCGTGTTTCAAGACGG-3′. The sequence of D1/D2 26S rDNA (accession numbers: MH754705.1) obtained above was aligned using BLAST analysis (http://blast.ncbi.nlm.nih.gov/Blast.cgi). The sequence which shared over 98% similarity with currently available sequence was considered to be the same species. The phylogenetic tree was constructed and visualized using Mega 7 software [34].

### Screening of the yeast strains for intracellular lipids and PEFA production

The nineteen *Rhodotorula* strains isolated (Additional file 1: Table S1) were cultivated in the YPD seed culture medium at 28 °C and 180 rpm for 16 h, and then 5 mL of each seed culture was inoculated into the 250-mL flask containing 30.0 mL of the PEFA screening medium. The flasks were aerobically incubated at 28 °C and 180 rpm for 144 h. The PEFA, intracellular lipid and cell dry weight were determined as described below.

### Microscopic visualization of PEFA and intracellular lipid

The mixture of yeast culture and Nile red solution (5 μg/mL in DMSO) was incubated at room temperature for 10 min. Visualization was performed under bright field mode and under fluorescence (530 nm excitation and 626 nm emission) at 100× magnification using a Olympus U-LH100HG fluorescent microscope. The images of the cells were recorded using the cell Sens Standard software.

### Optimization of the medium for PEFA production

The strain P4R5 was cultivated aerobically in the YPD seed culture medium at 28 °C and 180 rpm for 16 h. A total of 5.0 mL of the culture was inoculated into the 250-mL flask containing 30.0 mL of the PEFA production medium supplemented with different concentrations of glucose and inulin. The flasks were aerobically incubated at 28 °C and 180 rpm for 144 h. The PEFA, intracellular lipid and cell dry weight were determined as described below.

### Ten-liter batch fermentation

The fermentation for the PEFA and intracellular lipid production was carried out in a 10-L fermentor (BIOQ-6005-6010B, Huihetang Bio-Engineering Equipment (Shanghai) CO-LTD). The seed culture of the yeast strain P4R5 was prepared as described above and inoculated into 7.0 L of the optimized PEFA production medium with a 10% inoculation scale. The fermentation was performed under the conditions of the agitation speed of 300 rpm, the aeration rate of 600 l/h, the temperature of 28 °C and the fermentation period of 168 h. Throughout the whole fermentation period, 50.0 mL of the culture was collected in the interval of 12 h and used for the determining of the PEFA titer, the content of intracellular oil, inulinase activity, cell density, cell dry weight, and residual reducing sugar and total sugar.

### Inulinase activity assay

The inulinase activity in the supernatant obtained were determined by the method described previously [21]. One unit of inulinase activity was defined as the amount of enzyme that catalyzes the release of 1.0 μmol of reducing sugar per min.

### Determination of reducing sugar and total sugar

Reducing sugar was determined using the Nelson-Somogyi method [35]. Total sugar was measured using the same method as reducing sugar after acid hydrolysis with 6.0 M HCl.

### Extraction and quantitative measurements of extracellular PEFA, cell dry weight and intracellular lipids

The PEFA were extracted from the yeast culture according to the reported procedure [17]. Thirty milliliter of culture broth was centrifuged at 8000*g* for 10 min. The precipitate containing cells and extracellular PEFA were further washed twice with 50 mL of distilled water to remove all traces of culture elements, and then extracted twice with 40 mL of ethyl acetate for PEFA solubilization. The cell pellet and solubilized PEFA were separated by centrifugation at 8000×*g* for 10 min. The solvent containing PEFA was freeze-dried overnight, and the dry weight of the ethyl acetate extract residue was used to estimate grams of PEFA per liter culture. Additionally, the cell pellet was freeze-dried to measure dry cell weight. Subsequently, the obtain dry cell were used for intracellular lipids extraction according to a previous study [36]. Briefly, a known weight (about 100 mg) of dry cells were lysed in 300 μL 6 mol/L HCl at 100 °C for 5 min, followed by adding 900 μL of chloroform–methanol solution (2:1, v/v). The chloroform phase of the mix was subsequently transferred to a well-weighed glass vial, and vacuum dried at 50 °C for 30 min. After evaporation of the solvent, the residues were weighed, and the grams of intracellular lipids per gram dry cell weight were calculated.

### GC–MS analysis

For the analysis of the fatty acid composition of PEFA and intracellular oil, the samples extracted above were transmethylated according to the reported procedure [22]. The fatty acid methyl esters (FAMEs) obtained were determined via gas chromatography/mass spectrometry (GC/MS) using an Agilent 7890A gas chromatograph interfaced with an Agilent 5975C mass-selective detector as previously reported [22].

### HPLC analysis of polyols and acetic acid

One gram of the extracted PEFA was thoroughly hydrolyzed by the addition of 10.0 mL of 2.0 M NaOH solution. Subsequently, the mixture was extracted with chloroform to remove the generated fatty acid, and the aqueous phase containing the polyols was taken out for chromatographic analysis. HPLC was performed on an Agilent 1100 series HPLC system (Agilent Technologies, Palo Alto, CA, USA) equipped with an refractive index detector using an Aminex HPX-87C analytical column (300 × 7.8 mm) (Bio-Rad, USA). HPLC separation was carried out in a eluant of pure water with a flow rate of 0.3 mL/min. The acetic acid, sorbitol, xylitol, mannitol and arabitol bought from Sigma were used as the standards.

### LC–ESI–MS

LC–ESI–MS analysis of PEFA was performed on a Waters Acquity UPLC H-Class/SQD II system (Waters Corp., Milford, MA, USA), equipped with an ACQUITY UPLC BEH Shield RP18 (100 mm × 2.1 mm, 1.7 μm) column. The chromatography separation was performed as described by Guerfali et al. [17]. The LC–MS instrument was operated in positive ion electrospray mode with an acquisition range of *m/z* 115–1700 with a scan rate of 0.5 spectra/s.

### Surface tension measurement and critical micelle concentration (CMC)

The CMC of the PEFA was determined by using the surface tension measurement method [37]. The analysis was performed on a programmable tensiometer (Model K20 Easy Dyne, KRUSS GmbH, Germany) by the Du Nouy ring method under atmospheric pressure and temperature of 25 ± 1 °C. Surface tensions of the PEFA at the different concentrations ranging from 1 to 1440 mg/L were recorded until the data were constant. The platinum ring of the tensiometer was cleaned with acetone and flame-dried before each measurement.

### Emulsification index determination

Emulsification index (*E*_*24*_) towards diesel, as well as other hydrophobic substrates including n-hexane, *n*-heptane, *n*-dodecane, soybean oil and olive oil, was determined as described by Guerfali et al. [17]. The studied hydrocarbons was added to 20 mg/mL PEFA in a ratio of 1:1 and vortexed vigorously for 2 min. After 24 h of incubation, the height of the emulsified layer was measured and compared with the total height of the liquid layer and multiplied by 100 (*E*_*24*_). Tween 80 was used as a control.

### Biodiesel preparation and its properties analysis

Intracellular lipids extracted from the cells of the strain P4R5 were transformed to biodiesel according to the procedure described [24]. In brief, 2.5 L fermentation broth harvested at 156 h from inulin during 10-L batch fermentation in this study were used for intracellular lipids preparation according to the above method. Then, 50.0 mL of intracellular lipids obtained were mixed with 500 mL of 0.1 M H_2_SO_4_-methanol solution. The mixture was kept on a magnetic stirrer at 70 °C with mild mixing for 40 min. After centrifugation at 3000×*g* for 10 min, the organic phase (lower phase) was heated at 85 °C for 90 min and the residual organic compounds obtained were used as biodiesel. For properties analysis, average unsaturation (AU) was calculated from the fatty acids compositional profiles based on the equation (AU = ΣN × Ci, where N represents the number of carbon–carbon double bonds of unsaturated fatty acids and Ci represents the percentage of the component). Based on the value of AU, viscosity, specific gravity, cloud point, cetane, iodine number and higher heating value were calculated using the following equations: viscosity = − 0.6316 × AU + 5.2065, iodine value = 74.373 × AU + 12.71, Specific gravity = 0.0055 × AU + 0.8726, cetane = − 6.6684 × AU + 62.876, HHV = 1.7601 × AU + 38.534, and cloud point = − 13.356 × AU + 19.994 [32].

### Statistical analysis

All values were means of three separate experiments. The statistical analysis was performed using the GraphPad Prism 6.01 (GraphPad Software Inc., USA). Means were compared and analyzed using either t-test or one way analysis of variance (ANOVA) with Tukey HSD post hoc multiple comparison test. A probability value of *P* < 0.05 was considered significant.

## Supplementary information

**Additional file 1: Table S1.** The morphological and physiological characteristics, accession numbers of D1/D2 26S rDNA sequences, identified species, the biomass, intracellular lipids content and PEFA production in the PEFA screening medium of the 19 strains isolated from deep-sea.

## References

[1] Zaky AS, Tucker GA, Daw ZY, Du CY (2014). Marine yeast isolation and industrial application. FEMS Yeast Res.

[2] Nagahama T, Hamamoto M, Nakase T, Takami H, Horikoshi K (2001). Distribution and identification of red yeasts in deep-sea environments around the northwest Pacific Ocean. Antonie van Leeuwenhoek.

[3] Zhu ZW, Zhang SF, Liu HW, Shen HW, Lin XP, Yang F, Zhou YJJ, Jin GJ, Ye ML, Zou HF, Zhao ZBK (2012). A multi-omic map of the lipid-producing yeast *Rhodosporidium toruloides*. Nat Commun.

[4] Garay LA, Sitepu IR, Cajka T, Fiehn O, Cathcart E, Fry RW, Kanti A, Nugroho AJ, Faulina SA, Stephanandra S (2017). Discovery of synthesis and secretion of polyol esters of fatty acids by four basidiomycetous yeast species in the order Sporidiobolales. J Ind Microbiol Biotechnol.

[5] Zhuang X, Kilian O, Monroe E, Ito M, Tran-Gymfi MB, Liu F, Davis RW, Mirsiaghi M, Sundstrom E, Pray T (2019). Monoterpene production by the carotenogenic yeast *Rhodosporidium toruloides*. Microb Cell Fact..

[6] Banat IM, Franzetti A, Gandolfi I, Bestetti G, Martinotti MG, Fracchia L, Smyth TJ, Marchant R (2010). Microbial biosurfactants production, applications and future potential. Appl Microbiol Biotechnol.

[7] Pacwa-Plociniczak M, Plaza GA, Piotrowska-Seget Z, Cameotra SS (2011). Environmental applications of biosurfactants: recent advances. Int J Mol Sci.

[8] Marchant R, Banat IM (2012). Microbial biosurfactants: challenges and opportunities for future exploitation. Trends Biotechnol.

[9] Araujo HWC, Andrade RFS, Montero-Rodriguez D, Rubio-Ribeaux D, da Silva CAA, Campos-Takaki GM (2019). Sustainable biosurfactant produced by *Serratia marcescens* UCP 1549 and its suitability for agricultural and marine bioremediation applications. Microb Cell Fact..

[10] Marchut-Mikolajczyk O, Drozdzynski P, Pietrzyk D, Antczak T (2018). Biosurfactant production and hydrocarbon degradation activity of endophytic bacteria isolated from *Chelidonium majus* L. Microb Cell Fact..

[11] Cajka T, Garay LA, Sitepu IR, Boundy-Mills KL, Fiehn O (2016). Multiplatform mass spectrometry-based approach identifies extracellular glycolipids of the yeast *Rhodotorula babjevae* UCDFST 04-877. J Nat Prod.

[12] Garay LA, Sitepu IR, Cajka T, Xu J, Teh HE, German JB, Pan ZL, Dungan SR, Block DE, Boundy-Mills KL (2018). Extracellular fungal polyol lipids: a new class of potential high value lipids. Biotechnol Adv.

[13] Sen S, Borah SN, Bora A, Deka S (2017). Production, characterization, and antifungal activity of a biosurfactant produced by *Rhodotorula babjevae* YS3. Microb Cell Fact.

[14] Lyman M, Rubinfeld B, Leif R, Mulcahy H, Dugan L, Souza B (2018). *Rhodotorula taiwanensis* MD1149 produces hypoacetylated PEFA compounds with increased surface activity compared to *Rhodotorula babjevae* MD1169. PLoS ONE.

[15] Garay LA, Sitepu IR, Cajka T, Cathcart E, Fiehn O, German JB, Block DE, Boundy-Mills KL (2017). Simultaneous production of intracellular triacylglycerols and extracellular polyol esters of fatty acids by *Rhodotorula babjevae* and *Rhodotorula* aff. *paludigena*. J Ind Microbiol Biotechnol..

[16] Li YH, Zhao ZB, Bai FW (2007). High-density cultivation of oleaginous yeast *Rhodosporidium toruloides* Y4 in fed-batch culture. Enzyme Microb Technol.

[17] Guerfali M, Ayadi I, Mohamed N, Ayadi W, Belghith H, Bronze MR, Ribeiro MHL, Gargouri A (2019). Triacylglycerols accumulation and glycolipids secretion by the oleaginous yeast *Rhodotorula babjevae* Y-SL7: structural identification and biotechnological applications. Bioresour Technol.

[18] Tang RR, Chi Z, Jiang H, Liu GL, Xue SJ, Hu Z, Chi ZM (2018). Overexpression of a pyruvate carboxylase gene enhances extracellular liamocin and intracellular lipid biosynthesis by *Aureobasidium melanogenum* M39. Process Biochem.

[19] Huang C, Chen XF, Xiong L, Chen XD, Ma LL, Chen Y (2013). Single cell oil production from low-cost substrates: the possibility and potential of its industrialization. Biotechnol Adv.

[20] Liu GL, Chi Z, Chi ZM (2013). Molecular characterization and expression of microbial inulinase genes. Crit Rev Microbiol.

[21] Liu GL, Fu GY, Chi Z, Chi ZM (2014). Enhanced expression of the codon-optimized exo-inulinase gene from the yeast *Meyerozyma guilliermondii* in *Saccharomyces* sp. W0 and bioethanol production from inulin. Appl Microbiol Biotechnol..

[22] Shi NC, Mao WA, He XX, Chi Z, Chi ZM, Liu GL (2018). Co-expression of exo-inulinase and endo-inulinase genes in the oleaginous yeast *Yarrowia lipolytica* for efficient single cell oil production from inulin. Appl Biochem Biotechnol.

[23] Kurtzman C, Fell JW, Boekhout T (2011). The yeasts: a taxonomic study.

[24] Wang GY, Chi Z, Song B, Wang ZP, Chi ZM (2012). High level lipid production by a novel inulinase-producing yeast *Pichia guilliermondii* Pcla22. Bioresour Technol.

[25] Wang ZP, Fu WJ, Xu HM, Chi ZM (2014). Direct conversion of inulin into cell lipid by an inulinase-producing yeast *Rhodosporidium toruloides* 2F5. Bioresour Technol.

[26] Ageitos JM, Vallejo JA, Veiga-Crespo P, Villa TG (2011). Oily yeasts as oleaginous cell factories. Appl Microbiol Biotechnol.

[27] Van Bogaert INA, Holvoet K, Roelants SLKW, Li B, Lin YC, Van de Peer Y, Soetaert W (2013). The biosynthetic gene cluster for sophorolipids: a biotechnological interesting biosurfactant produced by *Starmerella bombicola*. Mol Microbiol.

[28] Aguilar-Osorio G, vanKuyk PA, Seiboth B, Blom D, Solomon PS, Vinck A, Kindt F, Wosten HAB, de Vries RP (2010). Spatial and developmental differentiation of mannitol dehydrogenase and mannitol-1-phosphate dehydrogenase in *Aspergillus niger*. Eukaryot Cell.

[29] Abbasi H, Hamedi MM, Lotfabad TB, Zahiri HS, Sharafi H, Masoomi F, Moosavi-Movahedi AA, Ortiz A, Amanlou M, Noghabi KA (2012). Biosurfactant-producing bacterium, *Pseudomonas aeruginosa* MA01 isolated from spoiled apples: physicochemical and structural characteristics of isolated biosurfactant. J Biosci Bioeng.

[30] Dhasayan A, Kiran GS, Selvin J (2014). Production and characterisation of glycolipid biosurfactant by *Halomonas* sp. MB-30 for potential application in enhanced oil recovery. Appl Biochem Biotechnol..

[31] Sajjadi B, Raman AAA, Arandiyan H (2016). A comprehensive review on properties of edible and non-edible vegetable oil-based biodiesel: composition, specifications and prediction models. Renew Sustain Energy Rev.

[32] Hoekman SK, Broch A, Robbins C, Ceniceros E, Natarajan M (2012). Review of biodiesel composition, properties, and specifications. Renew Sustain Energy Rev.

[33] Khot M, Kamat S, Zinjarde S, Pant A, Chopade B, RaviKumar A (2012). Single cell oil of oleaginous fungi from the tropical mangrove wetlands as a potential feedstock for biodiesel. Microb Cell Fact..

[34] Kumar S, Stecher G, Tamura K (2016). MEGA7: molecular evolutionary genetics analysis version 7.0 for bigger datasets. Mol Biol Evol..

[35] Marais JP, De Wit JL, Quicke GV (1966). A critical examination of the Nelson-Somogyi method for the determination of reducing sugars. Anal Biochem.

[36] Zhang H, Wang Z, Feng Y, Cui Q, Song X (2019). Phytohormones as stimulators to improve arachidonic acid biosynthesis in *Mortierella alpina*. Enzyme Microb Technol.

[37] Baruah A, Pathak AK, Ojha K (2015). Study on the thermal stability of viscoelastic surfactant-based fluids bearing lamellar structures. Ind Eng Chem Res.

