# Supplementary material for: Efficient simultaneous production of extracellular polyol esters of fatty acids and intracellular lipids from inulin by a deep-sea yeast Rhodotorula paludigena P4R5
Source: Microb Cell Fact. 2019 Sep 3;18:149. doi: 10.1186/s12934-019-1200-3 (PMC6720868; doi:10.1186/s12934-019-1200-3)
Supplement: Supplementary file 1 — Additional file 1: Table S1. The morphological and physiological characteristics, accession numbers of D1/D2 26S rDNA sequences, identified species, the biomass, intracellular lipids content and PEFA production in the PEFA screening medium of the 19 strains isolated from deep-sea. [file 12934_2019_1200_MOESM1_ESM.docx]

Table S1 The morphological and physiological characteristics, accession numbers of D1/D2 26S rDNA sequences, identified species, the biomass, intracellular lipids content and PEFA production in the PEFA screening medium of the 19 strains isolated from deep-sea

| Strains | Morphological and physiological characteristics^a^ | Accession numbers of D1/D2 26S rDNA sequences | Species | Biomass  (g/L) | Intracellular lipids content (w/w) | PEFA titer (g/L) |
| --- | --- | --- | --- | --- | --- | --- |
| P4R1 | Cells were subglobose or ovoid and spore-free. Yeast colonies were round, smooth, and orange. Reproduction occurred by multilateral or polar budding. Fermentative ability was absent. Cells could synthesize intracellular oils and red or yellow pigments. | MH753693.1 | *Rhodotorula mucilaginosa* | 16.27 | 46.4% | ND |
| P4R2 |  | MH753694.1 | *Rhodotorula sphaerocarpa* | 21.82 | 39.1% | ND |
| P4R3 |  | MH753695.1 | *Rhodotorula sphaerocarpa* | 20.62 | 41.8% | ND |
| P4R4 |  | MH753702.1 | *Rhodotorula diobovata* | 18.58 | 40.2% | 1.5 |
| P4R5 |  | MH753703.1 | *Rhodotorula paludigena* | 20.93 | 48.9% | 12.7 |
| P4R6 |  | MK386936.1 | *Rhodotorula mucilaginosa* | 18.81 | 41.2% | ND |
| P4R7 |  | MK386932.1 | *Rhodotorula mucilaginosa* | 17.50 | 47.0% | ND |
| P4R8 |  | MK386934.1 | *Rhodotorula mucilaginosa* | 19.07 | 49.4% | ND |
| P4R9 |  | MK386933.1 | *Rhodotorula mucilaginosa* | 20.11 | 49.3% | ND |
| P4R10 |  | MK386939.1 | *Rhodotorula slooffiae* | 13.59 | 42.1% | ND |
| P4R11 |  | MK386935.1 | *Rhodotorula mucilaginosa* | 21.62 | 49.9% | ND |
| P4R12 |  | MK377444.1 | *Rhodotorula mucilaginosa* | 18.31 | 41.9% | ND |
| P4R13 |  | MK377445.1 | *Rhodotorula mucilaginosa* | 19.61 | 41.14 | ND |
| P4R14 |  | MK386937.1 | *Rhodotorula mucilaginosa* | 20.75 | 19.11 | ND |
| P4R15 |  | MK377446.1 | *Rhodotorula mucilaginosa* | 22.26 | 22.08 | ND |
| P4R16 |  | MK379578.1 | *Rhodotorula mucilaginosa* | 21.45 | 19.01 | ND |
| P4R17 |  | MK379579.1 | *Rhodotorula mucilaginosa* | 22.16 | 16.16 | ND |
| P4R18 |  | MK379580.1 | *Rhodotorula mucilaginosa* | 22.53 | 20.30 | ND |
| P4R19 |  | MK379591.1 | *Rhodotorula mucilaginosa* | 21.96 | 16.81 | ND |

^a^All the strains were found to show the similar morphological and physiological characteristics.

The values were means of three independent determinations.

ND not detected.
